# Multiple Praziquantel Treatments of *Schistosoma mansoni* Egg-Negative, CCA-Positive Schoolchildren in a Very Low Endemic Setting in Egypt Do Not Consistently Alter CCA Results

**DOI:** 10.4269/ajtmh.18-0961

**Published:** 2019-04-22

**Authors:** Ayat A. Haggag, Miriam Casacuberta Partal, Amal Rabiee, Khaled M. Abd Elaziz, Carl H. Campbell, Daniel G. Colley, Reda M. R. Ramzy

**Affiliations:** 1 Ministry of Health and Population, Cairo, Egypt;; 2 Department of Parasitology, Leiden University Medical Center, Leiden, The Netherlands;; 3 Department of Community, Environmental and Occupational Medicine, Faculty of Medicine, Ain Shams University, Cairo, Egypt;; 4 Center for Tropical and Emerging Global Diseases, University of Georgia, Athens, Georgia;; 5 Department of Microbiology, University of Georgia, Athens, Georgia;; 6 National Nutrition Institute, General Organization for Teaching Hospitals and Institutes, Cairo, Egypt

## Abstract

Forty-four *Schistosoma mansoni* egg-negative/circulating cathodic antigen (CCA) low-positive (trace or 1+) children in three districts of very low prevalence in Egypt were given three sequential praziquantel (PZQ) treatments. Stool and urine specimens were collected 3 months following the initial treatment, and 3 weeks following the second and following the third PZQ treatments, which were conducted 5 weeks apart. Stool specimens were examined by Kato–Katz (four slides/stool sample) and all *S. mansoni* egg-negative stools were further tested by the “miracidia hatching test” (MHT). Urine samples were examined by the point-of-care CCA assay (POC-CCA). Over the study period, all stool samples from study subjects remained *S. mansoni* egg negative and MHT negative. Of the POC-CCA test results, in the first day of the study 3 months following the initial treatment, 29.5% were negative, 61.4% CCA trace positives, and 9.1% CCA 1+ positives. Following each PZQ treatment, the test results fluctuated between 1+, trace, and negative, but did not consistently decrease. The proportions of POC-CCA–positive results obtained in the first day (70.5%) as compared with the last day of the study (72.7%) in all of the three districts were very similar. We conclude that CCA trace and 1+ readings, in Kato–Katz *S. mansoni* egg-negative children in this area with very low levels of intestinal schistosomiasis, are not consistently altered or rendered consistently negative following repeated PZQ treatments and are therefore likely to represent false-positive readings. This finding is of critical importance for countries such as Egypt as they approach elimination.

## INTRODUCTION

Schistosomiasis (also known as bilharzia) is a waterborne parasitic disease endemic in 78 tropical and subtropical countries worldwide. The disease is caused by an infection with blood flukes of *Schistosoma* spp. and is transmitted to humans through transcutaneous penetration by its larval stages following human direct contact with infested water.^1^ By 2016, it was estimated that at least 206.4 million people required preventive treatment for schistosomiasis.^2^ Successful schistosomiasis control programs in Japan, China, Brazil, and Egypt have shown that progression toward elimination of schistosomiasis through persistent control is feasible.^3^ In some areas, the global emphasis is now shifting from control to elimination of schistosomiasis.^4^

Successful schistosomiasis control programs, based on preventive chemotherapy using praziquantel (PZQ),^5^ have significantly decreased disease endemicity. Consequently, to overcome the drawbacks of the Kato–Katz stool examination in the diagnosis of *Schistosoma mansoni* infection, especially in low endemicity areas,^6^ several programs have used a commercial point-of-care circulating cathodic antigen (POC-CCA) test. Several studies conducted in low prevalence areas have observed POC-CCA–positive persons who are *S. mansoni* egg negative by the Kato–Katz assay.^7–9^ Because this group (Kato–Katz–negative/POC-CCA–positive individuals) represents a challenge for programs shifting from control to elimination (interruption of transmission), we carried out an in-depth study of this particular group.^10^ In that study, we followed a cohort of 45 schoolchildren residing in a very low *S. mansoni* prevalence area for 30 successive days. Over the 30 days, only 0.07% of stool samples were *S. mansoni* egg and “miracidia hatching test” (MHT) positive. However, most of urine POC-CCA assays (89.1%) remained positive at the trace or 1+ level. These findings indicate that these children are not likely to be a threat to themselves in terms of ongoing egg-focused morbidity or to continued transmission. However, the trace and 1+ POC-CCA results, which may or may not be false positives, were a cause for further study. It could be hypothesized that these are not false positives and that additional treatment with PZQ would lead to their changing to negatives. In this case, the low-level POC-CCA readings would indicate that these children still harbored a few viable adult worms, perhaps secreting low levels of CCA antigens.

In the present report, we address this hypothesis by questioning whether the trace and 1+ CCA readings would change to negative following repeated standard PZQ treatments. We have now followed a cohort of 44 schoolchildren (of the 45 previously studied) through three rounds of PZQ treatments by assaying their stools and urines for three successive days following each of the three PZQ treatments.

## MATERIALS AND METHODS

### Ethics statement.

The Ethics Review Committee of the Ministry of Health and Population reviewed and approved the study protocol (number 9-2018/4). The Institutional Review Board of the University of Georgia (UGA) evaluated the protocol and determined UGA personnel to be not engaged. The children were enrolled in the study after obtaining written informed consent from their parents/guardians. The study objectives, the need for treatment, and follow-up of stool and urine samples were explained to the children and their parents/guardians. The work included only noninvasive collections of stool and urine specimens, and standard treatment with PZQ. Provision of stool and urine samples was taken as children’s assent.

### Study design and subjects.

**Figure 1.**
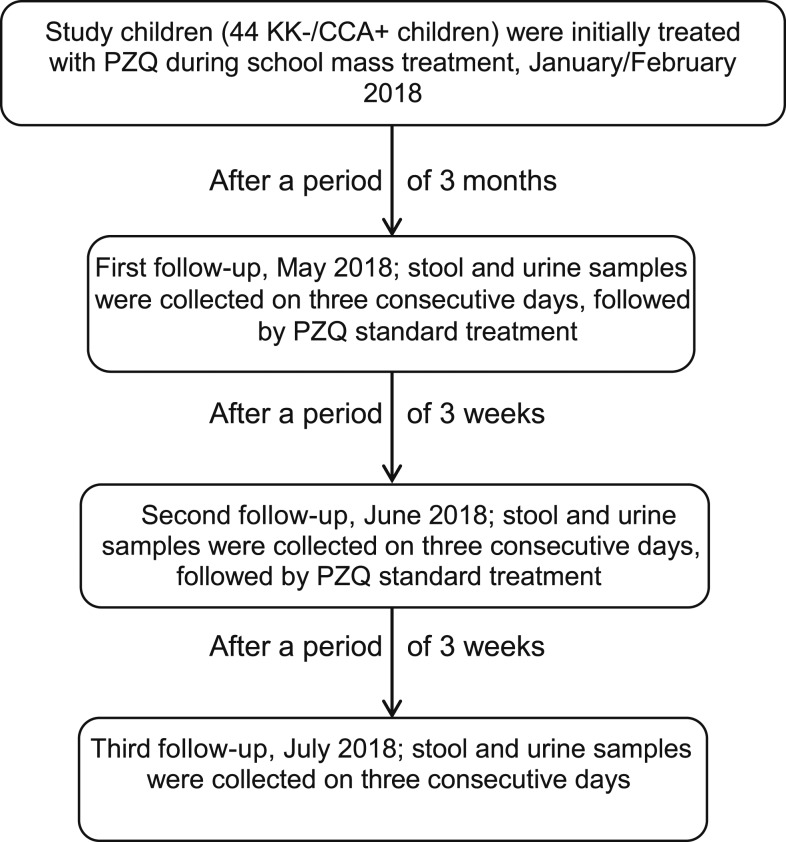
Flow diagram of the follow-up protocol following three rounds of PZQ treatment. At each survey time point, three stool and three urine samples were collected on consecutive days and were examined by Kato–Katz and POC-CCA assay, respectively. POC-CCA = point-of-care circulating cathodic antigen; PZQ = praziquantel.

The study design is shown in Figure 1. The study was conducted in January/February, May, June, and July 2018 and included a cohort of 44 schoolchildren attending schools in three districts (Al Riad, Desouk, and Sidy Salem districts) in Kafr El Sheikh Governorate, Egypt. The 44 schoolchildren participated in a previous 30-day follow-up study by our group^10^ and were treated with a single regimen of PZQ (40 mg/kg) after completion of that study, during mass treatment of their schools, conducted during January–February 2018. Three months later in May 2018, stool and urine samples were collected from these 44 schoolchildren on three successive days and examined. Each stool sample was examined by Kato–Katz (four slides/stool sample), and all *S. mansoni* egg-negative samples by Kato–Katz were also examined by the MHT. Each urine sample was assayed by the POC-CCA (one urine/one POC-CCA). Each of the 44 schoolchildren were then treated a second time (May 2018) and a third time (June 2018), each time with 40 mg/kg of PZQ. Three weeks after each of these treatments, each of the 44 schoolchildren had stool and urine samples collected on three successive days in June and July, respectively, and assayed in the same way by Kato–Katz and POC-CCA.

### Kato–Katz thick smear technique.

The Kato–Katz stool analysis was performed according to the WHO standard procedure.^11^

### Point-of-care circulating cathodic antigen assay.

The POC-CCA test (batch number: 170914118; expiration date: 9/2019) was performed according to the manufacturer’s instruction (Rapid Medical Diagnostics, Pretoria, South Africa). Briefly, two drops of urine were added to the sample well of the cassette and allowed to absorb completely into the specimen pad. The test was read after 20 minutes; any line in the test area was considered positive, and the band density was recorded. In reading and scoring the POC-CCA results, we used 10 (G1–G10) “standardized POC-CCA cassettes” graded G1 (negative test) to G10 (strong positive). The relationship between the G1–G10 grading scale and the more standard evaluations of negative, trace, 1+, 2+, and 3+ was described previously.^10^ Briefly, G1 indicates no observable CCA band; that is, a negative POC-CCA; band intensities of G2 and G3 are considered trace readings, whereas band intensities of G4 and G5 are considered 1+ readings. The test was considered invalid if the line developed after 25 minutes or no control line developed. The test was read and agreed on by two observers (laboratory assistant or laboratory technician), and in case of disagreement, results were discussed and resolved by a senior laboratory technician.

### The MHT.

This technique was performed as previously described.^12^ Briefly, from each stool sample, a portion of about 3 gm was emulsified in 75 mL of physiological saline and sieved rapidly through five successive standard brass sieves (mesh openings 2,000, 500, 212, 125, and 32 μm, respectively) by spraying dechlorinated water from a 2-L plastic spray bottle. Any material remaining on the upper surface of the last, smallest, mesh-opening sieve was washed with dechlorinated water into a 1-L clear conical flask, which was covered completely with a black cloth except the small neck, and filled with dechlorinated water (pH 7.4–7.6) to the rim. The flask was left strongly illuminated from one side at room temperature. After 2, 4, and 6 hours, each flask rim was examined for swimming miracidia using a handheld lens. Observed miracidia indicated a positive MHT, that is, presence of viable *S. mansoni* eggs in the stool sample. The absence of miracidia indicated a negative MHT, that is, no viable, mature *S. mansoni* eggs in the stool sample assayed.

### Statistical analyses.

Data entry was performed on Microsoft Excel database spreadsheet and the descriptive data are presented. Although three stool and three urine specimens were obtained on three consecutive days at each parasitologic survey time point in this research study, we herein report most of the data based on the assays performed on the first day’s specimens at each time point. This emphasis on the first day’s specimens is because most neglected tropical disease programs would likely only collect one specimen for programmatic surveys.

## RESULTS

### Praziquantel treatments.

A total of 44 schoolchildren (14 from Al Riad, and 15 each from Desouk and Sidy Salem district, Kafr El Sheikh Governorate) were enrolled in this study and received three successive treatments with PZQ (Figure 1). The cohort comprised 17 males and 27 females and they ranged from 8 to 15 years of age (mean age: 11.1 ± 1.6 years). All study children were directly observed to have ingested the drug tablets at each treatment, and they reported that they had had no activity in or contact with fresh water possibly infested with schistosome larvae starting in January 2018 (because of cold weather) and during the period of the study.

### Parasitological examination.

The study included three follow-up parasitological evaluations, and at each follow-up evaluation time point, a stool sample was collected on each of three successive days (Figure 1). Throughout the study, all Kato–Katz examinations and all MHT assays were uniformly negative from all of the schoolchildren.

### Point-of-care circulating cathodic antigen testing.

**Figure 2.**
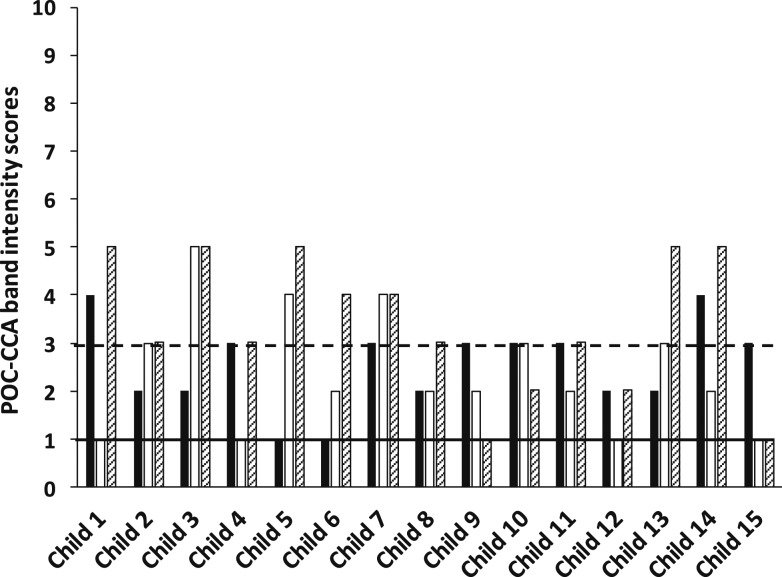
Point-of-care circulating cathodic antigen results of 15 children studied in Desouk district, following three rounds of praziquantel treatment. Urines were collected from 15 children 3 months after their initial treatment with PZQ and assayed by POC-CCA (black bars). They were then treated a second time with PZQ, and 3 weeks later, their urines were collected and assayed by POC-CCA (open bars). They were then treated a third time with PZQ, and 3 weeks later, their urines were collected and assayed by POC-CCA (hatched bars). The numbers on the *Y* axis denote the band intensity readings on the G1 to G10 scale. The horizontal black line is at a reading of one, which indicates no observable CCA band, therefore a negative POC-CCA. The horizontal dashed line is set to mark the limit of trace readings (band intensities of two and three), greater than which are considered 1+ readings (band intensities of four and five). At each time point, urines were collected and assayed on three consecutive days, but only the first day’s results are plotted in this graph. POC-CCA = point-of-care circulating cathodic antigen; PZQ = praziquantel.

**Figure 3.**
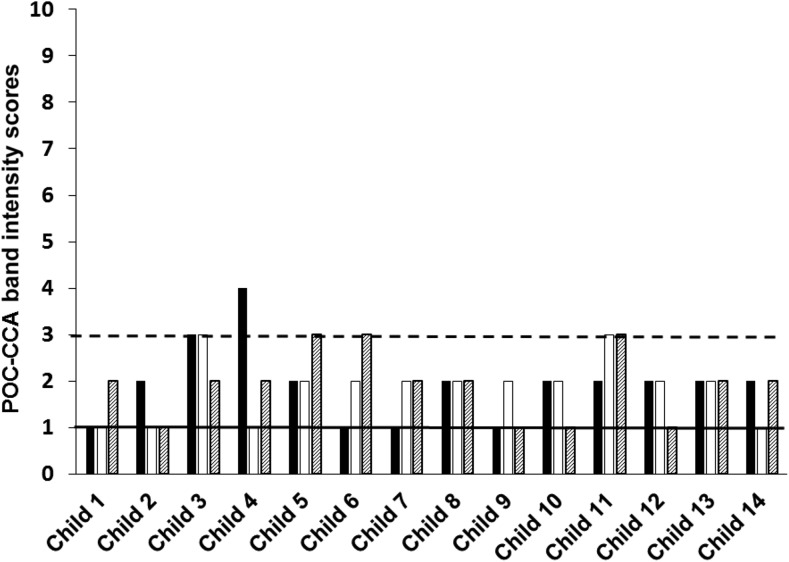
Point-of-care circulating cathodic antigen results of 14 children studied in Al Riad district, following three rounds of praziquantel treatment. Urines were collected from 15 children 3 months after their initial treatment with PZQ and assayed by POC-CCA (black bars). They were then treated a second time with PZQ, and 3 weeks later, their urines were collected and assayed by POC-CCA (open bars). They were then treated a third time with PZQ, and 3 weeks later, their urines collected and assayed by POC-CCA (hatched bars). The numbers on the *Y* axis denote the band intensity readings on the G1 to G10 scale. The horizontal black line is at a reading of one, which indicates no observable CCA band, therefore a negative POC-CCA. The horizontal dashed line is set to mark the limit of trace readings (band intensities of two and three), greater than which are considered 1+ readings (band intensities of four and five). At each time point, urines were collected and assayed on three consecutive days, but only the first day’s results are plotted in this graph. POC-CCA = point-of-care circulating cathodic antigen; PZQ = praziquantel.

**Figure 4.**
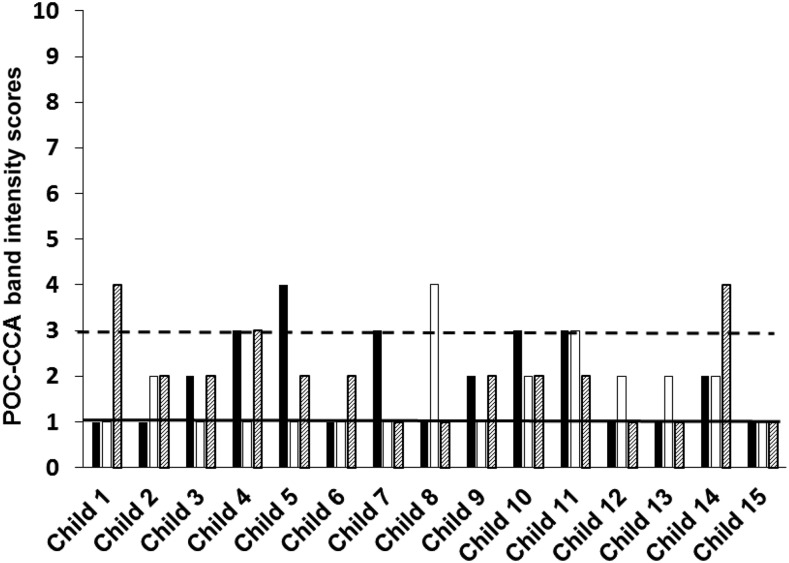
Point-of-care circulating cathodic antigen results of 15 children studied in Sidy Salem district, following three rounds of praziquantel treatment. Urines were collected from 15 children 3 months after their initial treatment with PZQ and assayed by POC-CCA (black bars). They were then treated a second time with PZQ, and 3 weeks later, their urines were collected and assayed by POC-CCA (open bars). They were then treated a third time with PZQ, and 3 weeks later, their urines collected and assayed by POC-CCA (hatched bars). The numbers on the *Y* axis denote the band intensity readings on the G1 to G10 scale. The horizontal black line is at a reading of one, which indicates no observable CCA band, therefore a negative POC-CCA. The horizontal dashed line is set to mark the limit of trace readings (band intensities of two and three), greater than which are considered 1+ readings (band intensities of four and five). At each time point, urines were collected and assayed on three consecutive days, but only the first day’s results are plotted in this graph. POC-CCA = point-of-care circulating cathodic antigen; PZQ = praziquantel.

As with the parasitological testing, at each of the three follow-up survey times, a urine specimen was collected on each of the three successive days and each urine specimen was tested by a POC-CCA assay (Figure 1). As shown in Table 1, the POC-CCA test results of the first day of the follow-up evaluation 3 months after the initial treatment were 13 (29.5%) CCA negatives, 27 (61.4%) CCA trace positives, and four (9.1%) CCA 1+ positives. The number of POC-CCA positives (31 [70.45%]) recorded on the first day of the initial evaluation following the first treatment was very similar to that recorded on the first day of the last evaluation (32 [72.7%]), following the last of the three treatments. Thus, overall, there was no difference between the mixture of POC-CCA results recorded on the first day of the study for the three districts as compared with the last day of the study, following three PZQ treatments: 12 (27.3%) CCA negatives, 26 (59.1%) CCA trace, and six (13.6%) CCA 1+. Table 1 compares these POC-CCA results recorded for the first and last days of the study for each district. Figures 2–4 compare the POC-CCA results of the first day of each follow-up for each child in Desouk, Al Riad, and Sidy Salem districts, respectively, following each of the three PZQ treatments. The POC-CCA test results fluctuated irregularly between being 1+, trace, and negative.

**Table 1:** Comparison of the first POC-CCA readings recorded for the 44 schoolchildren 3 months following the first treatment (first day) and 3 weeks following the third treatment (last day) of the study presented by district

District	Day of study	POC-CCA scores		
		G1 (negative)	G2–G3 (trace)	G4–G5 (1+)
		No. (%)	No. (%)	No. (%)
Desouk (*n* = 15)	First day	2 (13.3)	11 (73.4)	2 (13.3)
	Last day	4 (26.7)	7 (46.6)	4 (26.7)
Al Riad (*n* = 14)	First day	4 (28.6)	9 (64.3)	1 (7.1)
	Last day	3 (21.4)	10 (71.5)	1 (7.1)
Sidy Salem (*n* = 15)	First day	7 (46.7)	7 (46.7)	1 (6.7)
	Last day	5 (33.3)	9 (60.0)	1 (6.7)
All districts (*n* = 44)	First day	13 (29.5)	27 (61.4)	4 (9.1)
	Last day	12 (27.3)	26 (59.1)	6 (13.6)

POC-CCA = point-of-care circulating cathodic antigen.

G1 indicates no observable CCA band, that is, a negative POC-CCA.

G2 and G3 band intensities are considered trace readings.

G4 and G5 band intensities are considered 1+.

It can be seen in Figures 2–4 that there were several negative POC-CCA readings for the first urine evaluations at the last time points. Figures 2–4 graph data from the first of three urines at each of the three posttreatment surveys. By this presentation, there is only one child (representing 2.3% of the group of 44) who was actually CCA negative for all three urine specimens after the second and third PZQ treatments. However, when evaluating all three urine POC-CCA readings obtained at the time of the last survey, there were three children (6.8%) who had negative CCA readings for all three of their last urine specimens. Nevertheless, none of the test results of the other 41 children (93.2%) consistently became negative following multiple PZQ treatments. Based on the continued variation we observed across the three urines from these 41 children at the final survey, the several negatives on the first urines appear to represent the consistently observed variation from 1+ to trace to negative.

## DISCUSSION

This study was designed to answer an important question related to the specificity of the POC-CCA assay in very low prevalence of *S. mansoni*–endemic areas. This is particularly important if the CCA urine assay is to be used to guide intervention choices as overall prevalence declines and elimination of *S. mansoni* transmission appears feasible. If the trace and 1+ CCA readings we reported previously^10^ were because of viable adult worms present in the study children secreting low levels of CCA antigens, such readings would be expected to have changed to consistently negative following repeated treatments with standard doses of PZQ.

Our finding that there was no difference in the ratio of POC-CCA–positive results in the study children on the first day of the study (3 months after an initial treatment with PZQ) compared with the last day of the study (after these three cycles of PZQ treatment), (70.45% versus 72.7%), clearly indicates that the trace and 1+ CCA readings are very unlikely to be due to viable *S. mansoni* worms. Accordingly, the specificity of the trace CCA bands observed for these Kato–Katz–negative children is more likely lower than those observed in higher prevalence *S. mansoni*–endemic settings.

In a previous study carried out by Mwinzi et al.,^7^ one or two PZQ treatments converted most Kato–Katz–negative/POC-CCA–positive individuals to POC-CCA negativity. Schoolchildren enrolled in their study were from an area of 10–15% prevalence by the Kato–Katz method. Note that according to a report by the WHO,^13^ an area with a prevalence less than 10% and an intensity of infection < 100 eggs per gram of feces should be considered a low endemic zone. In our study, however, the 44 schoolchildren were selected from three districts, Desouk, Al Riad, and Sidy Salem, where the overall prevalence of *S. mansoni* infection, as determined by the Kato–Katz stool examination,^8^ was very low (1.2%, 0%, and 0.9%, respectively).^10^ Thus, the findings of our study add significant new information of particular programmatic interest in such very low prevalence areas.

We did see that three children had negative CCA readings for all three of their last three urine specimens. Although the general inconsistency of the CCA readings of negative, trace, and 1+ might argue against firm conclusions regarding these three children, it is certainly possible that one or more of these children (6.8% of those studied) had a few worms that were eventually killed by the multiple treatments. However, given the repeated fluctuations of urine readings between negative and 1+ by most children (93.2%), it is just as likely that conversion to negative by these three children was due to chance readings.

To the best of our knowledge, no previous formal study has addressed the performance of the POC-CCA assay in such a very low *S. mansoni*–endemic setting, following years of persistent programmatic control measures.^8^ Additional studies in other areas of very low prevalence and intensity of *S. mansoni* infection should be encouraged. In addition, new sensitive and specific diagnostic tools, such as the up-converting phosphor lateral flow circulating anodic antigen assay, possibly through specimen pooling strategies,^14^ will be needed as more country programs move from control to elimination (interruption of transmission) of *S. mansoni*.

In conclusion, the present findings support our previous proposition that children who are Kato–Katz negative/POC-CCA trace or 1+, and reside in areas with such low prevalence, are not likely to be a threat to themselves, nor are they likely to contribute to substantial continued transmission.^10^ Furthermore, the variability and lack of turning consistently negative after multiple treatments make it likely that the trace and 1+ CCA readings in such areas are false positives.
